# Curriculum Innovations: Teaching Residents About Guideline Development and Cost-Conscious Care: A Mixed-Method Evaluation Study

**DOI:** 10.1212/NE9.0000000000200198

**Published:** 2025-02-28

**Authors:** Marian Majoie, Anne Visser, Merve Ulubas, Ghislaine van Mastrigt, Pablo Alonso-Coello, Claudia Valli, Ena Niño de Guzman, Gerald Gartlehner, Barbara Nussbaumer-Streit, Irma Klerings, Roberta James, Duncan Service, Silvia Evers, Jos Kleijnen, Maurizio Leone, Danielle Verstegen

**Affiliations:** 1 Academic Center of Epileptology Kempenhaeghe Maastricht UMC+, the Netherlands;; 2 Department of Psychiatry and Neuropsychology, Maastricht University, Faculty of Health, Medicine and Life Sciences, Mental Health and Neuroscience Research Institute (MHeNS), Maastricht, the Netherlands;; 3 Maastricht University, Faculty of Health, Medicine and Life Sciences, School of Health Professions Education (SHE), Maastricht, the Netherlands;; 4 Department of Neurology, Groene Hart ziekenhuis, Gouda, the Netherlands;; 5 Department of Neurology, Ziekenhuis Rivierenland Tiel, the Netherlands;; 6 Department of Health Services Research (NL), Maastricht University, Faculty of Health, Medicine and Life Sciences, Care and Public Health Research Institute (CAPHRI), the Netherlands;; 7 Iberoamerican Cochrane Centre, Sant Antoni Maria Claret, Barcelona, Spain;; 8 Institut de Recerca Sant Pau (IR SANT PAU), Barcelona, Spain;; 9 Centro de Investigación Biomédica en Red de Epidemiología y Salud Pública (CIBERESP), Madrid, Spain;; 10 Department for Evidence-based Medicine and Evaluation, University for Continuing Education Krems, Austria;; 11 SIGN Healthcare Improvement Scotland, Edinburgh;; 12 Kleijnen Systematic Reviews Ltd (UK); and; 13 Fondazione IRCCS Casa Sollievo della Sofferenza, San Giovanni Rotondo, Italy.

## Abstract

**Introduction and Problem Statement:**

Clinical practice guidelines (CPGs) can play a crucial role in achieving better and more cost-efficient health care. This requires training health care providers in CPG development. This study presents results of design-based research on a training program based on the learning theory of problem-based learning, following the underlying principles of active, authentic, collaborative, and blended learning.

**Objectives:**

The CoCoCare training aims to equip neurology residents with insight in CPG development (including cost aspects) and understanding of how to apply CPGs in clinical practice.

**Methods and Curriculum Description:**

The CoCoCare training consisted of 6 asynchronous e-learning modules, synchronous workshops (1 day onsite in run 1 and dispersed online sessions in run 2), and a series of online assignments mimicking the steps of CPG development. Participants could present their draft guidelines at the European Academy of Neurology conference. Between 2019 and 2021, 55 participants from 21 European member states participated in the training. A mixed-method design-based research study design was used to collect questionnaire and interview data from participants and trainers.

**Results and Assessment Data:**

Participants felt that the training prepared them to participate in CPG development and that they had learned how to critically appraise a guideline and were stimulated to apply guidelines in practice. Trainers were also positive and observed an upward trend in the knowledge of the participants. Participants especially appreciated the active (assignments) and authentic learning in the practical part of the training, but the workload of the assignments in combination with clinical work was a challenge. They also enjoyed the collaborative learning in an international group of residents but asked for more interaction and support during the period dedicated to assignments.

**Discussion and Lessons Learned:**

The CoCoCare training program proved to be a feasible and unique, international guideline development training with a focus on cost-efficient care. Recommendations include to arrange accreditation, ensure regular interaction between trainers and participants, review the workload of assignments, and look at possibilities to provide feedback. Future research should evaluate the impact on CPG application and participation in CPG development.

## Introduction

Our health care system is not equipped to meet the needs of patients in future decades. Delivered care is fragmented, collaboration between professionals is suboptimal, access to health care is under pressure, and clinicians face work dissatisfaction.^1-3^ If we do nothing, globally health care costs will double by 2040, and health care will become unaffordable.^4^ Therefore, health care should focus on improving population health, optimizing costs, enhancing patient experience, and improving work-life balance for clinicians.^5^

Clinical practice guidelines (CPGs) are statements that include recommendations intended to optimize patient care that are informed by a systematic review of evidence and an assessment of the benefits and harms of alternative care options. In addition, they consider contextual factures such as costs, feasibility, and equity. CPGs can make an important contribution because they bring together scientific knowledge, leadership, professionalism, legislation, regulations, and patient perspectives. As such, they have a considerable influence on the quality and effectiveness of health care and, once they incorporate costs and health-related outcomes (e.g., health benefit and quality-of-life effect), allow (future) health care professionals to become cost-conscious.^6^

Clinical practice guidelines are developed on a (sub)national level with limited standardization across organizations and fields. This causes differences in treatment decisions and, therefore, treatment quality and effectiveness among countries and institutions.^7-9^ CPGs are intended to guide various, often multidisciplinary, decision-making processes and to enable treatment teams to find a balance between quality, effectiveness, and costs. There is a perceived need for institutions to improve knowledge of CPGs, evidence on costs, and inclusion of quality-adjusted life year measurements.^6,10,11^ Also, despite the major effect these guidelines can have on the behavior of health care professionals,^12^ we rarely see their development, application, and implementation addressed in (para)medical training programs or education.

Based on the perceived needs of health care professionals, we developed a training program to facilitate cost-conscious quality care. The cost-conscious care or CoCoCare training is a state-of-the-art educational program to be used for medical residents. The objective of the CoCoCare training is two-fold: (1) to teach residents the basics of developing and implementing high-quality, evidence-based guidelines and (2) to equip residents with the competences to work in a cost-conscious manner. These objectives are addressed by a blended learning program that combines e-learning, workshops, self-study, and assignment work. The content of the program covers all steps that are crucial in developing CPGs: evidence-based decision making, high-quality patient-centered care, cost-conscious care, and implementation in medical practice.

The design of the CoCoCare training is based on the learning theory of problem-based learning, following the underlying principles of active, authentic, collaborative, and blended learning,^13^ although not in the traditional format because of the blended learning nature of the program. To stimulate understanding and transfer of knowledge, participants actively engaged in assignments derived from the practice of CPG development, with exercises mimicking the steps of guideline development subsequently applied in developing their own (first draft of a) guideline. Deep learning is further stimulated by organizing discussions with peers during (onsite or online) workshops and peer collaboration working on assignments. Additional study materials using authentic examples were provided in e-learning modules.

This study aims to evaluate the design of the CoCoCare training and to gain insight into whether it achieves the intended outcomes. The goal of the evaluation is to gain insight into the experiences of all stakeholders and to further improve the design of the CoCoCare training. The research question is how can a training based on principles of active, authentic, collaborative, and blended learning support residents to gain insight into CPG development (including cost aspects) and understanding of how to apply CPGs in clinical practice?

## Methods

### Study Design

The CoCoCare evaluation is set up as a mixed-method study in the framework of design-based research.^14^ The rationale for a mixed-method study design is to improve future trainings by using qualitative feedback and to strengthen the external validity of our findings. Design-based research focuses on the development of (educational) interventions, which reflect on theory, by directly investigating their effect in practice and revising in an iterative cycle. Therefore, this study is aimed at gaining insight into the effectiveness of the educational design of the training based on principles of active, authentic, collaborative, and blended learning. Both quantitative and qualitative results were collected from trainers and participants, and results were repeatedly discussed in the whole research team to ensure contribution and insights from different perspectives.

### Training Intervention

The CoCoCare training consisted of 3 parts: e-learning modules, onsite and online workshops, and online assignments. The asynchronous e-learning was delivered through an online learning platform and mainly included video presentations and quizzes. It served as an introduction to the CoCoCare training and preparation for the workshops. Each module was designed to take a maximum of 2 hours to complete.

In the first pilot run, a workshop day was organized at the European Academy of Neurology (EAN) conference. It consisted of face-to-face lectures and practical exercises for all topic areas: evidence-based decision-making, high-quality patient-centered care, cost-conscious care, and implementation in medical practice. In the second run, which took place during the COVID-19 pandemic, this workshop day was replaced by separate online workshops that were integrated into the period that participants worked on assignments. After the workshops, participants worked on a series of assignments over several months. During this period, the participants received online training assignments related to the guideline development process. The assignments included viewing a slidecast (for example, about the basic terms used in health economic evaluation), reading articles, answering questions in short quizzes (e.g., which tools are used in implementation), and participating in discussions at the CoCoCare forum. The main assignment was to identify a clinical problem in the field of neurology for which a guideline had to be developed, develop a PICO (patient, intervention, control, outcome) question, and perform a literature study using specific tools such as the GRADE methodology. While the assignments were created by the CoCoCare trainers, the participants' local supervisors were supposed to help them complete the assignments. The final assignment of the training was a poster presentation of the guideline the participants had developed during this period. These guidelines were based on a diagnosis and/or treatment of a neurologic disorder and were newly developed.

The training aimed to achieve the following learning objectives:

The participants should:

- Understand the value and the limitations of guidelines.
- Understand how to apply guidelines.
- Have insight in how guidelines are developed.
- Have insight in the different aspects that are considered when a guideline is developed (including cost considerations).
- Be able to participate in a committee that develops a new guideline.

A summary of the curriculum design and content is presented in Table 1. In addition, in conclusion, the training was executed 2 times with slight variations:

- Run 1 (pilot); one year, part-time: 6 asynchronous e-learning modules (designed to last 2 hours each) and an onsite workshop day (consisting of 4 workshops) at the EAN conference in 2019, followed by online assignments with very limited synchronous contact. Participants were provided with the opportunity to present the draft guideline that they developed at the online EAN conference in 2020.
- Run 2; one year, part-time: 6 asynchronous e-learning modules, biweekly 1-hour online workshops, and assignments. Workshops were split up, provided online, and dispersed in-between assignment work. Participants were provided with the opportunity to present the draft guideline that they developed at the online EAN conference in 2021.

**Table 1:** Curriculum Design and Content

0. Precourse meeting	
	During this meeting, trainers will be introduced and the outline of the training will be presented
1. Introduction to guideline development	
	This will be a general introduction to guidelines. To help clinicians make evidence-based medical decisions, guideline developers often grade the strength of their recommendations and rate the quality of the evidence informing those recommendations. Participants will also learn how to judge the quality of guidelines (AGREE II checklist)
2. From the question to the certainty of evidence	
• Developing a research question and outcome ranking • Risk of bias, hierarchy of the evidence, and summary of findings table	A guideline panel should define the scope of the guideline and the planned recommendations. Each recommendation should answer a focused and sensible health care question that leads to an action. Guideline developers must, and authors of systematic reviews are strongly encouraged to, specify all potential patient-important outcomes as the first step in their endeavor. Guideline developers will also make a preliminary classification of the importance of the outcomes. The end point for systematic reviews and for HTA restricted to evidence reports is a summary of the evidence, the quality rating for each outcome, and the estimate of effect. For guideline developers and HTA that provide advice to policymakers, a summary of the evidence represents a key milestone on the path to a recommendation
3. Cost-conscious care	
	Providing care that is of high value to the patient but also cost effective needs to consider economic aspects and quality-of-life outcomes. The participants will learn to methodologically develop, teach, and study the functioning of the health care system from an economic perspective
4. Implementation in medical practice	
	In the implementation of clinical guidelines, feasibility, practicality, uptake, and acceptance play important roles. The participants will learn to develop tools and methods to improve the implementation of guidelines. Moreover, they will be supported in personal development to implement the guidelines themselves, including decisiveness and leadership
5. Evidence to decision	
	To come from scientific evidence to a concise and acceptable recommendation, several aspects should be taken into consideration. Participants will learn how an evidence-to-decision table integrates all aspects of guideline development to subsequently formulate a recommendation that is acceptable to each stakeholder.

### Participants

Participants were residents in neurology, approached through the EAN by using their website and email lists. The European Academy of Neurology has an extensive network of residents affiliated with it. This is the Resident and Research Fellow Section.^15^ They were informed about the training by email and were invited to apply. Interested parties sent a curriculum vitae and a motivation letter. Because the interest was greater than the number of participating places, a group of participants was selected based on their motivation and available time to follow the course. Owing to the involvement of the EAN with its extensive multinational network, it was more feasible to primarily recruit neurology residents.

### Trainers

When working out the Erasmus grant application, we went looking for experts in the field of guideline development who were also active in the field of training. These partners are ultimately also the ones who either gave the training themselves or put forward people from their department to give training.

### Data and Analysis

For the evaluations, a combination of instruments was used to collect quantitative and qualitative data:

- Questionnaires about the training early on, midterm, and at the end of training (both participant groups)
- The Maastricht High Value Cost-Conscious Care Attitude Questionnaire measuring attitudes regarding cost-conscious care,^16^ at the start and at the end (only second run)
- Group and individual interviews (designers/trainers and participants)
- Written feedback by email (designers/trainers)

Questionnaires and interview guides were purposively developed to probe experiences and insights regarding the learning objectives and the training design, more specifically the aspects of active, authentic, collaborative, and blended learning.

Questionnaire data were collected using Qualtrix and analyzed using descriptive statistics in Excel. Semistructured group or individual interviews were conducted, onsite when possible, and otherwise online: group interviews with participants of cohort 1 at the end of the workshop day (by the first author), individual online interviews with participants of cohort 2 (by the second and third author), and brief group interviews with participants at the end of the last online workshop (by the first and last author). Most of the designers of the training were also involved as trainers; therefore, we considered them as one group. Interviews with designers/trainers were performed during project meetings, when possible, roughly half-way and at the end of the training (by the last author). When this was not possible, designers/trainers were invited for an individual online meeting or they provided written input by email. These data were aimed to collect information to improve the training design. Therefore, interviews were not fully transcribed, but summaries of experiences and suggestions were made by the interviewers and sent back to the participants for member check. Interviews with designers/trainers were analyzed by the last author and then discussed by the entire team during project meetings. For the interviews of training participants, we chose 2 residents who performed all tasks, 2 who performed less than 50% of all tasks (43% of tasks), and a resident who did not perform any of the tasks. Unfortunately, we did not receive a response from the resident who did not perform any of the tasks. We used standardized questions, and the interviews were anonymized and thematically analyzed by the second and third author; emerging themes were discussed with the first and last author.

### Ethical Considerations

Participation in the CoCoCare training was voluntary and free of cost. Participation in the evaluation study was voluntary; participants provided informed consent and were free to withdraw at any time without affecting their participation in the training itself. Ethical permission for this evaluation study was obtained from the Maastricht University Faculty of Health, Medicine and Life Sciences Research Ethics Committee (FHML-REC), under number FHML-REC/2019/034. Changes in the planned procedure were approved under number FHML-REC/2019/034/Amendment1.

### Reflexivity

The authors contributed to the research from different backgrounds and expertise. All authors were involved in the CoCoCare project. The first author (M.M.) is a physician involved in guideline development, D.V. is an educational scientist who has expertise in training design but is not involved in guideline development, and A.V. and M.U. are physicians involved in guideline development. All other authors/trainers were experienced guideline developers and members of professional guideline organizations (e.g., GIN, GRADE, Cochrane, and NICE).

### Data Availability

Data not provided in this article because of space limitations may be shared (anonymized) at the request of any qualified investigator for purposes of replicating procedures and results.

## Results

We present aggregated results of the participants' and designers'/trainers' evaluations here. A more detailed description can be found in the CoCoCare evaluation report.^17^

### Participants

The first pilot run of the CoCoCare training was conducted from June 2019 until June 2020. Twenty-two residents in neurology from 16 countries participated. Five participants presented the guidelines that they developed during the course at the online EAN conference in 2020.

The second run was postponed because of COVID-19 regulations and conducted from September 2020 until June 2021. 33 residents in neurology from 14 different countries participated. 13 participants presented the guidelines they developed at the online EAN conference in 2021. An overview of this is presented in Table 2, and a selection of the presented guidelines can be found in Ref. 18.

**Table 2:** Overview on Time Frame, Participants, and Presentations

	Time frame	Participants	European countries	Guideline presentations during EAN
First run (pilot)	June 2019–June 2020	22 neurology residents	16	5
Second run	September 2020–June 2021	33 neurology residents	14	13

### Training Participants: Evaluation

#### First Pilot Cohort

All 22 residents filled out the questionnaire after completing the e-learning modules and the onsite workshop day. Table 3 provides the results of the questionnaire completed at the end of the workshop day. A focus group discussion was also held at the end of the workshop day. The participants valued the e-learning modules, but they preferred the interactive workshops. Points of interest identified in the questionnaire were the time that participants reported to have spent on the e-learning (varying from 2 hours to several days) and the intensity of the workshop day. Participants were not really sure what to expect of the assignments following in the period after the workshops and would have liked more information earlier on.

**Table 3:** Participants' Evaluation of the CoCoCare e-Learning Modules and Workshops: First Run (Pilot)

Item (5-point scale from 1 = strongly disagree to 5 = strongly agree)	Average	SD
1. During the CoCoCare training I learned a lot	4.7	0.5
2. The content of the CoCoCare training suited my needs	4.6	0.6
3. The CoCoCare training has encouraged me to apply guidelines	4.5	0.7
4. The CoCoCare training has encouraged me to reflect on my role as a doctor in developing guidelines	4.6	0.7
5. The CoCoCare training has encouraged me to reflect on my role as a doctor in providing cost-conscious care	4.4	0.7
6. The e-learning modules were useful to me	4.6	0.6
7. The e-learning modules were engaging	4.5	0.7
8. The e-learning modules were an adequate preparation for the workshops	4.5	0.7
9. The workshop “Evidence-based decision making” was useful to me	4.5	0.6
10. The workshop “High quality patient-centered care” was useful to me	4.6	0.6
11. The workshop “Cost-conscious care” was useful to me	4.3	0.7
12. The workshop “Implementation in medical practice” was useful to me	4.4	0.7
13. The workshop trainers were competent	4.9	0.4
14. The examples and exercises used in the e-learning modules and workshops were realistic	4.6	0.6
15. I know what will be expected from me in the CoCoCare follow-up with assignments	4.0	0.8
16. I feel adequately prepared for the CoCoCare follow-up with assignments	4.0	0.8
17. Working on this topic in a group has added value to me	4.5	0.6
18. The combination of training formats (e-learning, workshops, follow-up with assignments) is suitable	4.8	0.4

Participants‘ evaluation of the CoCoCare e-learning modules and workshops (n = 22).

Note that the assignments were called “on-the-job training” for participants; for consistency reasons, we have changed this to “assignments” here.

Eleven participants filled out the questionnaire at the end of the CoCoCare training. The results of the second questionnaire are provided in Table 4. Participants were positive about the CoCoCare training content and format. They felt especially stimulated to apply guidelines and reflect on their own (future) role in developing guidelines and in providing cost-conscious care. The feasibility, clarity, and relevance of the assignments scored slightly lower than the e-learning modules (e.g., feasibility of the assignments: average 4.1, SD 0.8; feasibility of the e-learning: average 4.6, SD 0.7). Most of the respondents asked for more interaction and support during the period that they were doing assignments, for example, in regular workshops or online discussion meetings.

**Table 4:** Participants' Evaluation of the CoCoCare Training as a Whole: First Run (Pilot)

Item (5-point scale from 1 = strongly disagree to 5 = strongly agree)	Average	SD
1. During the CoCoCare training I learned a lot	4.5	0.7
2. The content of the CoCoCare training suited my needs	4.4	0.7
3. The CoCoCare training has encouraged me to apply guidelines	4.5	0.7
4. The CoCoCare training has encouraged me to reflect on my role as a doctor in developing guidelines	4.6	0.5
5. The CoCoCare training has encouraged me to reflect on my role as a doctor in providing cost-conscious care	4.6	0.5
6. I have applied what I learned in the CoCoCare training in my daily work	4.1	0.6
7. The examples and exercises used in the e-learning modules and workshops were realistic	4.2	0.8
8. Working on this topic in a group has added value to me	4.2	0.8
9. The combination of training formats (e-learning, workshops, follow-up with assignments) is suitable	4.4	0.5
10. The e-learning modules were useful to me	4.6	0.7
11. The face-to-face workshops were useful to me	4.7	0.5
12. The follow-up with assignments were useful to me	4.1	0.8
13. The follow-up assignments were clear	3.6	0.9
14. The follow-up assignments were relevant to me	3.7	0.7
15. The follow-up assignments were engaging	4.0	0.5
16. The follow-up assignments were feasible	3.3	0.7
17. The CoCoCare learning environment provided enough support during the CoCoCare training	4.1	0.8
18. My supervisor supported me during the CoCoCare training	3.8	0.8

Participants' evaluation of the CoCoCare training at the end (n = 11 for items 1–5; n = 9 for items 6–18).

Note that the assignments were called “on-the-job training” for participants; for consistency reasons, we have changed this to “assignments” here.

#### Second Cohort

Nine participants filled out the questionnaire at the end of the e-learning modules, and 13 returned the questionnaire at the end of the CoCoCare training. Tables 5 and 6 present evaluation data of the second cohort. The respondents were very positive about the e-learning modules (average 4.7, SD 0.5), the teachers (that they met in online workshops), the quality of the learning materials, and the insight in how guidelines are developed. Similar to the first cohort, participants noted that they were not sure on what to expect from the assignments. Suggestions related mostly to practicalities, such as the timing of workshops. Regarding the results of the Maastricht High Value Cost-Conscious Care Attitude Questionnaire, definite conclusions could not be made because of a low number of respondents. However, there was a trend toward more acceptance of taking into account cost aspects in patient care.

**Table 5:** Participants' Evaluation of the CoCoCare e-Learning Modules: Second Run

Item (5-point scale from 1 = strongly disagree to 5 = strongly agree)	Average	SD
1. During the CoCoCare e-learning I learned a lot	4.6	0.5
2. The content of the CoCoCare e-learning suited my needs	4.4	0.5
3. The CoCoCare e-learning has encouraged me to apply guidelines	4.6	0.5
4. The CoCoCare e-learning has encouraged me to reflect on my role as a doctor in developing guidelines	4.6	0.5
5. The CoCoCare e-learning has encouraged me to reflect on my role as a doctor in providing cost-conscious care	4.7	0.5
6. The e-learning modules were useful to me	4.7	0.5
7. The e-learning modules were engaging	4.3	0.7
8. The examples and exercises used in the e-learning modules were realistic	4.6	0.7
9. I know what will be expected from me in the CoCoCare follow-up assignments	3.9	0.6
10. I feel adequately prepared for the CoCoCare follow-up assignments	3.9	0.9
11. The combination of training formats (e-learning, follow-up assignments) is suitable	4.2	0.7

Evaluation data from training participants after the e-learning modules (n = 9).

Note that the assignments were called “on-the-job training” for participants; for consistency reasons, we have changed this to “assignments” here.

**Table 6:** Evaluation Results of the CoCoCare Training as a Whole: Second Run

Item (5-point scale from 1 = strongly disagree to 5 = strongly agree)	Average	SD
1. During the CoCoCare training I learned a lot	4.7	0.5
2. I have applied what I learned in the CoCoCare training in my daily work	3.9	0.5
3. The combination of training formats (e-learning, follow-up assignments) is suitable	4.2	0.8
4. The e-learning modules were useful to me	4.7	0.5
5. The follow-up assignments were useful to me	4.4	0.7
6. The follow-up assignments were relevant to me	4.4	0.7
7. The follow-up assignments were engaging	4.5	0.7
8. The CoCoCare learning environment provided enough support during the CoCoCare training	4.3	0.6
9. My supervisor supported me during the CoCoCare training	3.8	1.3

Evaluation results of the CoCoCare training at the end (n = 12; excluding the respondent who did not answer the evaluation items).

Note that the assignments were called “on-the-job training” for participants; for consistency reasons, we have changed this to “assignments” here.

At the end of the second run, 4 participants were interviewed online. They all stated that the CoCoCare experience was enjoyable and a unique chance to learn about a topic they did not know much about. They mentioned that they learned how to evaluate the cost aspects of care. The interviewees were positive about the format of the course, and they felt that the practical part, that is, applying what they had learned in the assignments, was important. They also valued the virtual interaction with other participants, because of the positive vibe and ability to network. They would have preferred to have some face-to-face meetings besides the online workshops. The participants mentioned that it was a challenge to combine their clinical work with the CoCoCare training, but they felt that they were well informed about the required investment upfront.

### Designers/Trainers

In the first pilot cohort, the designers/trainers were content with the e-learning modules, although they felt that the technical quality varied and they identified some topics (in their own field of expertise) that they felt needed to be covered more extensively than possible within the time limitations agreed beforehand. They were very positive about the onsite workshops: participants were enthusiastic, and the atmosphere was good. However, they felt that 1 day was too short to conduct all 4 workshops. Better alignment of the different workshops and the e-learning modules was needed.

During the period dedicated to assignments, the designers/trainers noticed that the assignments were too much work for some of the participants. They thought that the participants needed more information early on and more support during this part of the training. Their impression was that local supervisors were hardly involved, and they believed that one reason for this might be that the role of the local supervisor was not explicitly described in the CoCoCare materials. Finally, they felt that they had lost contact with the participants after the workshops.

In the second cohort, the trainers involved were the same as in the first cohort. They commented that they had enjoyed the interaction with participants during the workshops that were online and interspersed with the assignments in this run. It was good to see participants interact, to stay in contact with participants when they were making assignments, and to see what they had learned. The online workshops did add to the workload of trainers, that is, making it harder to contact with participants and assess how well everyone had understood what was told, but it was doable and they would like to keep this in future runs. They suggested reviewing the content of the e-learning and the online workshops to see whether this could be redistributed: some of this content could be moved to the assignment period (as e-learning or in the workshops). The trainers also commented, however, that the face-to-face workshops in the first run had advantages as well: direct interaction between trainers and participants and a valuable networking opportunity.

The trainers did notice a decline in participation toward the end of the assignment period. Only a minority of participants completed the last tasks. It would be good to look for ways to stimulate participation further. Suggestions were to look at the timing of (online) workshops and the format, possibly allowing more time for interaction with the trainers and actively tracking completion of assignments.

## Discussion

The CoCoCare training was developed to teach participants the basics of developing and implementing high-quality, evidence-based guidelines and to equip participants with the competences to work in a cost-conscious manner. The evaluation of this training through design-based research provided insight into the experiences of all stakeholders and resulted in suggestions to further improve the CoCoCare training.

The evaluation showed that the objectives were met. Participants felt able to perform critical appraisal on a guideline, to apply a guideline in practice, to provide cost-conscious care, and to participate in developing a guideline in the future. Trainers were also positive about the learning they observed: they saw an upward trend in the knowledge of the participants, and they noticed that their questions were more detailed. However, both participants and trainers felt that the distribution of content over the upfront e-learning modules, the (onsite and/or online) workshops, and the assignments could be improved.

Participants especially appreciated the active (assignments) and authentic learning in the practical part of the training, that is, to answer a unique clinical question during the course. The workload of the assignments in combination with COVID-19–related factors was, however, a problem for many participants and probably the main reason for dropout. Ways to stimulate participation to the end of the course could be to reduce the number of assignments, provide feedback on assignments, and arrange formal accreditation of the course. The participants also enjoyed the collaborative learning in an international group of residents. Apart from the opportunity to learn about guidelines, the course was also seen as a unique opportunity to network. Therefore, a combination of onsite workshops with online workshops is suggested for future runs.

A previous study using a competency-based medical education framework identified 3 core competencies for a guideline developer: facilitating the development of guideline structure and setup, making judgments about the quality or certainty of the evidence, and transforming evidence into a recommendation.^19^ These competencies help to standardize the qualifications needed for individuals to serve on guideline panels in varying capacity. During the CoCoCare training, awareness of and insight into these competencies were emphasized in the workshops and assignment work as the first step toward potential participation in CPG development in future but also as a way to stimulate CPG application in practice.

There are other dedicated trainings aimed at training guideline developers.^20^ The CoCoCare training aims at a different target group: residents who all need to understand the basics of guideline development to be able and motivated to apply guidelines in their clinical work. Some will hopefully become motivated to engage in subsequent training to become guideline developers.

Strengths of this study are that the design of the CoCoCare training is based on evidence-based learning theory and that the training was implemented and systematically evaluated in practice in an international environment with trainers and participants from a large number of European countries. We also implemented consecutive versions of the training, which allowed an evaluation of different formats and ways to offer the training in the future. A limitation is that we have not measured the impact with tests or observations of changed behavior. Moreover, all the evaluation results may be biased because it is likely that the most active participants reacted and filled out the questionnaires and most of the others did not. The participants did not receive any form of compensation and joined the course voluntarily. This shows their interest in the topic but may also have been a reason for dropout. It is not unreasonable to assume that residents prioritized other (mandatory) work. Furthermore, trainers evaluating their own work may have been potential bias. Trainers may experience challenges of keeping their teaching materials up to date. Finally, these training runs were executed during the COVID-19 pandemic, and this may have influenced the participation and results.

The CoCoCare training proved to be feasible for its participants, which implies that a similar training could improve clinical practice in a variety of fields. After further refinement and evaluation, we believe that the training could be relevant to a broader group of health care professionals, such as other specialists, researchers, and policymakers. Recommendations include to arrange accreditation, ensure regular interaction between trainers and participants, review the workload of assignments, and look at possibilities to provide feedback. Future research should evaluate the impact on CPG application and participation in CPG development.

In conclusion, we believe that the CoCoCare guideline training, which is based on principles of active, authentic, collaborative, and blended learning, succeeded in meeting the learning objectives of applying a guideline in practice, performing critical appraisal on a guideline, providing cost-conscious care, and participating in developing a guideline in the future.

Our design-based research study provided us with concrete suggestions for improvements for future runs, such as spreading of e-learning modules, onsite and online workshops, and the assignments.

**Take Home Messages**

→ A training for residents that is based on exercises that mimic clinical practice guideline (CPG) development is an effective way to raise awareness of the importance of CPG.
→ Involvement in (real or for practice) CPG development gives residents insight into different aspects that are considered when a guideline is developed (including cost aspects).
→ Training residents in CPG will likely contribute to CPG implementation and raise participation in CPG development groups.

## Glossary

CPGs: clinical practice guidelines
EAN: European Academy of Neurology
